# AC:DC – Agiles und kollaboratives digitales Klassenzimmer

**DOI:** 10.1365/s40702-021-00736-w

**Published:** 2021-05-12

**Authors:** Linda Grogorick, Jens Lamprecht

**Affiliations:** Lehrstuhl Informationsmanagement, Institut für Wirtschaftsinformatik, Technische Universität Braunschweig, Mühlenpfordtstr. 23, 38106 Braunschweig, Deutschland

**Keywords:** Digitales Lernen, Kooperation, Kollaboration, Lernerfolg, E‑Services, Digital Learning, Cooperation, Collaboration, Learning Success, E‑Services

## Abstract

Die Möglichkeiten zur Gestaltung digitaler Lehr- und Lernkonzepte sind vielfältig und mit verschiedenen Potenzialen verbunden. Trotz zahlreicher Bestrebungen digitale Bildungsangebote vermehrt an Hochschulen, Schulen und im Arbeitskontext zu etablieren, hat erst die rasante Ausbreitung von Covid-19 zu einem weitrechenden Umdenken geführt. In diesem Beitrag wird daher das Konzept AC:DC zum digitalen, agilen und kollaborativen Lernen vorgestellt, welches im Sommersemester 2020 erstmals in einer Mastervorlesung eingesetzt wurde. Die Evaluation der Lehrveranstaltung hat überwiegend positive Resultate gezeigt, vor allem in Hinblick auf den erreichten Lernerfolg.

## Abstract (en)

The possibilities for designing digital teaching and learning concepts are diverse and associated with various potentials. Despite numerous efforts to increasingly establish digital education opportunities at universities, schools, and in the workplace context, only the rapid spread of Covid-19 has led to a far-reaching rethinking. This paper therefore presents the AC:DC concept for digital, agile, and collaborative learning, which was used for the first time in a master’s lecture in the summer semester of 2020. The evaluation of the course has predominantly shown positive results, especially related to the achieved learning success.

## Einleitung und Motivation

Die Möglichkeiten zur Gestaltung des Lernens sind in der heutigen Zeit vielfältiger als noch vor einigen Jahren. Vor allem ist dies auf eine Digitalisierung des Lernens und Lehrens zurückzuführen, was mit zahlreichen Vorteilen einhergeht. Beispielsweise wird ein zeit- und ortsunabhängiges Lernen ebenso ermöglicht, wie ein individuelles oder gemeinsames kreatives und vor allem aktives Arbeiten, um sich neue Kenntnisse und Fähigkeiten anzueignen. Eine Unterstützung des individuellen und gemeinsamen Lernprozesses ist digital gezielter und vielleicht sogar besser möglich, wodurch sich Lernende insbesondere selbstständig Wissen aneignen können. Dies lässt die Bedeutung des selbstgesteuerten Lernens weiter ansteigen (Leimeister und David 2019). Zwar wächst die Bedeutung und der Einsatz des digitalen Lernens und Lehrens sowohl im Schul‑, Hochschul, als auch Arbeitskontexts stetig, doch ist eine flächendeckende sinnvolle Verbreitung trotz vielfältiger Initiativen noch längst nicht erreicht.

Mit der rasanten Ausbreitung von Covid-19 und zum Schutz vor einer Infektion prägt die Digitalisierung zunehmend die Art des Lernens und Lehrens. Digitale Bildung rückt demnach immer mehr in den Fokus und sorgt dafür, dass Lernen zunehmend digital verläuft, statt wie bisher weitestgehend analog. Dies beweisen auch die Ergebnisse des statistischen Bundesamts aus der Erhebung zur Nutzung von Informations- und Kommunikationstechnologien in privaten Haushalten. Digitale Lernangebote werden im ersten Quartal 2020 von 10- bis 15-jährigen Schüler/-innen mehr als sieben Mal so viel zur Kommunikation mit Lehrenden und anderen Lernenden genutzt als im ersten Quartal des Vorjahres. Ein ähnliches Ergebnis zeigt auch die Verwendung digitaler Lernangebote von Schüler/-innen und Studierenden ab 16 Jahren, wonach sich der Anteil im Vergleich zum Vorjahr immerhin verdoppelt hat (Statistisches Bundesamt 2020).

Vor diesem Hintergrund sind in den vergangenen Monaten auch zahlreiche neuartige Konzepte zum digitalen Lernen entstanden. In diesem Beitrag wird ein solches neuartiges Lehrkonzept für eine Vorlesung an einer Hochschule vorgestellt, wobei das digitale, agile und kollaboratives Lernen im Mittelpunkt steht.

## Kollaboratives und agiles digitales Lernen

Im wissenschaftlichen Diskurs zu Lehr- und Lernkonzepten stehen sich häufig kompetitive und kooperative sowie kollaborative Elemente gegenüber. Bei einem Wettbewerb treten Lernende gegeneinander an und konkurrieren um das Erreichen der bestmöglichen Leistung. Kooperatives Lernen hingegen beruht auf einer gegenseitigen Unterstützung der Lernenden, um zu einem gemeinsamen Lernziel zu gelangen (Eckardt und Finster 2019). Eine spezielle Form der Kooperation nimmt Kollaboration ein. Bei dieser wird „mit einem gemeinsamen Ziel gemeinsam an einem ‚Werkstück‘“ gearbeitet (Robra-Bissantz und Siemon 2019, S. 10). Diese zwei Formen der Zusammenarbeit beruhen aber beide darauf, dass Lernende sowohl innerhalb einer Gruppe als auch zwischen mehreren Lerngruppen miteinander interagieren und kommunizieren (Siemon et al. 2017).

Kollaboratives Erlernen von Kenntnissen und Fähigkeiten schafft, insbesondere in Verbindung mit einer ausgeprägten Vernetzung untereinander, eine gegenseitige Unterstützung beim Lernen, was wiederum die Entstehung einer Gemeinschaft fördert, die schlussendlich den Lern- und Studienerfolg positiv beeinflusst (Kallookaran und Robra-Bissantz 2016). Auch bei einem Vergleich von kollaborativen und kompetitiven Elementen erzielt nach Meinung von Studierenden eine Zusammenarbeit bessere Ergebnisse bezüglich des erreichten Lernerfolgs (Eckardt und Finster 2019). Trotz dieser Vorteile ist eine Förderung von Kollaboration bislang noch nicht konsequent genug in der Lehre integriert.

Eine Möglichkeit der Einbindung bietet das agile kooperative Lernen. Dabei erarbeiten Studierende in kurzen Zeiträumen eigenverantwortlich, sowie kooperativ in Gruppen, die Lernziele zu bestimmten Themenfeldern und erkennen jeweils neue. Lehrpersonen stehen dabei begleitend aber vor allem beratend zur Seite und stellen notwendige Lernmaterialien als Basiswissen digital zur Verfügung (Meissner und Stenger 2015).

Durch dieses Vorgehen sollen Studierende verschiedene Kompetenzen erwerben. Zunächst einmal steht dabei das eigenverantwortliche Erlernen von Fachwissen und Methoden durch ein selbstständiges Erarbeiten und kritisches Auseinandersetzen in Gruppen mit einer unbekannten Problemstellung im Fokus. Aber auch Teamfähigkeit und Sozialkompetenz wird durch eine Interdisziplinarität der Studierendengruppen gefördert. Darüber hinaus erwerben Studierende Kompetenzen in Hinblick auf Präsentationsfähigkeit und wissenschaftliches Arbeiten, welches gleichzeitig strukturiert und auch agil in einer digitalen Umgebung erfolgt.

Kollaboration und Agilität können insbesondere digital ermöglicht werden. Durch den Einsatz digitaler Plattformen sind Lerninhalte zeit- und ortsunabhängig verfügbar, was ein selbstbestimmtes Lernen zu jeder Zeit fernab des Vorlesungssaals unterstützt (Leimeister und David 2019). Vor diesem Hintergrund wird für das vorgestellte Lehr- und Lernkonzept dieses Beitrags eine kooperative agile Online-Plattform aufgebaut, die semesterweise mit neuen Inhalten erweitert wird, in der die Studierenden im Laufe des Semesters gemeinsam immer neue Problemstellungen bearbeiten und sich miteinander austauschen. Dadurch soll einerseits Interaktivität und Eigenverantwortung gefördert werden sowie andererseits über die Zusammenarbeit unter den Studierenden sowohl in der eigenen Gruppe als auch zwischen den Gruppen das Zusammengehörigkeitsgefühl untereinander erhöht werden, da ein kollaboratives Lernen positive Auswirkungen auf den Lern- und Studienerfolg hat (Dillenbourg 1999; Dillenbourg et al. 2009).

Durch die inhaltliche Verzahnung verschiedener digitaler Lehr- und Lernmethoden zu einem ganzheitlichem Lehrkonzept wird für die Studierenden insgesamt ein deutlich höherer Wert geschaffen, als es die Lehr- und Lernmethoden einzeln bieten können. Insbesondere die Integration der unterschiedlichen Ansätze stellt hierbei eine besondere Herausforderung dar, um den Lernenden ein aufeinander abgestimmtes Lernerlebnis zu bieten ohne Systembrüche oder -wechsel.

Bevor die Realisierung des neuen digitalen Lernangebots vorgestellt wird, beschreibt das nächste Kapitel zuerst den bereits seit mehreren Jahren erfolgreich durchgeführten Ablauf der Vorlesung, bevor dieser als reine digitale Lösung angepasst wurde.

## Status Quo der Vorlesung „E-Services“

Die Vorlesung „E-Services“ richtet sich an Masterstudierende verschiedener Studiengänge (z. B. Wirtschaftsinformatik oder Wirtschaftsingenieurwesen) und beschäftigt sich mit dem Charakter und Ausprägungsformen elektronischer Dienstleistungen in Business-to-Business- und Business-to-Consumer-Beziehungen. Studierende lernen zunächst die Besonderheiten der Dienstleistung, insbesondere digitaler Dienstleistungen, kennen. Daran knüpft sich eine Betrachtung des Werts von Dienstleistungen an und die differenzierte Betrachtung von diesem nach einer klassischen güterdominierten Logik und einer modernen servicedominierten Logik. Dies führt zur Gestaltung von Dienstleistungen sowie dem damit verbundenen Wert und der Integration von IT in den Dienstleistungsprozess. Im Anschluss erfolgt eine Betrachtung von Service-Netzwerken, die sich um eine Dienstleistung herum bilden sowie bei der Erbringung dieser mitwirken und so schlussendlich Service-Ökosysteme schaffen. Den Abschluss der Lehrveranstaltung bildet die Betrachtung von digitalen Plattformen, die zumeist das Zentrum von Service-Ökosystemen bilden und die Plattform-Technologien, die solche Plattformen erst ermöglichen.

Bei der Vermittlung der Lerninhalte wurde schon immer ein Fokus auf die Interaktion mit und zwischen den Studierenden gelegt. Zu diesem Zweck wurden Konzepte des Flipped Classrooms auf die Veranstaltung übertragen. Die Wissensvermittlung erfolgt dabei aus einer Kombination aus traditioneller Frontalveranstaltung und Videos zum selbstgesteuerten Erlernen der Inhalte. Begleitend erarbeiten die Studierenden in Kleingruppen zu jeder Vorlesungseinheit ein bestimmtes Thema, tragen dieses in einer späteren Veranstaltung vor und versuchen die anderen Studierenden einzubeziehen sowie eine Diskussion anzuregen, beispielsweise über kleine Quizze.

Bedingt durch die Maßnahmen zur Eindämmung und zum Schutz vor Covid-19 ergeben sich jedoch erhebliche Einschränkungen für das zuvor beschriebene Lehr- und Lernkonzept, da insbesondere die Arbeit in Kleingruppen und die gegenseitige Wissensvermittlung in Präsenzterminen nicht mehr möglich ist. Mit der Idee des *Agile and Collaborative Digital Classrooms (AC:DC)* ist ein digitales Lehr- und Lernkonzept entwickelt worden, das die vorhandenen Lerninhalte in ein digitales Format überträgt, aber nicht auf die interaktiven Elemente der bisherigen Veranstaltung verzichtet.

## Agile and Collaborative Digital Classroom

### Lehr- und Lernkonzept

Bedingt durch Covid-19 konnte die Vorlesung „E-Services“ als Präsenzveranstaltung in der Form nicht mehr durchgeführt werden. Für eine Fokussierung auf Digitalisierung wurde eine entsprechende Plattform zur Unterstützung des kollaborativen agilen Lernprozesses für Lernende und Lehrende aufgebaut. Dieser bietet Möglichkeiten zum Wissensaustausch und zur nachvollziehbaren Dokumentation zum Erhalt des Wissens für nachfolgende Lern-Generationen. Studierende bearbeiten im Laufe des Semesters immer neue Problemstellungen, tauschen sich miteinander aus und bearbeiten gemeinsam Themen.

„Agile and Collaborative Digital Classroom (AC:DC)“ ermöglicht in der Vorlesung „E-Services“ eine flexible und praxisnahe Lehre, in dem Digitalisierung mit Kooperation und agilem Lernen verbunden wird. Ein Wechsel aus Lerneinheiten und dem gemeinsamen aktiven Anwenden des Gelernten auf der digitalen Plattform durch Pitches, Diskussionen und Quizze prägen das Lehr- und Lernkonzept. Die Agilität des Lehrangebots zeichnet sich insbesondere durch die flexible Gestaltung der Lerninhalte aus. Die Studierenden übernehmen Eigenverantwortung und organisieren sich selbst, indem sie sich zunächst Basiswissen, das durch die Lehrenden zur Verfügung gestellt wird, aneignen und dann schauen, wie sie das Gelernte auf ein selbst gewähltes Praxisbeispiel übertragen sowie die Umsetzung planen und durchführen. Sollten die Studierenden hierbei weitere Lerninhalte benötigen, die nicht Teil des Basiswissens sind, können sie dies an die Lehrenden herantragen und werden dann bei der selbstständigen Erarbeitung dieser Inhalte begleitet und unterstützt, z. B. durch die Bereitstellung von ausgewählter Einstiegsliteratur. Nach jedem Pitch erhalten die Studierenden Feedback durch die anderen Studierenden aber auch durch die Lehrenden, wodurch das bereits angewandte Gelernte noch einmal reflektiert wird, was wiederum in Impulsen für den nächsten Pitch mündet. Durch die digitale Dokumentation auf der Plattform steht den Studierenden auch das Gelernte der bereits vergangenen Semester zur Verfügung und sorgt für Impulse, die das gesammelte Wissen iterativ praxisnäher und anschaulicher machen, so dass langfristig die Qualität steigt.

Die Inhalte der Vorlesung stehen auf Stud.IP Courseware als einzelne **Nano-Lerneinheiten** verständlich aufbereitet zum Selbststudium zur Verfügung. Jede Nano-Lerneinheit umfasst einen Einstiegsbereich, der zunächst mit einem so genannten Thought Starter das Thema einführt. Mithilfe eines anschaulichen Beispiels, einer Statistik oder einem Gimmick soll somit das Interesse geweckt werden. Weiterhin beinhaltet der Einstiegsbereich die festgelegten Lernziele, damit die Studierenden wissen, welche Kenntnisse und Fähigkeiten sie nach Abschluss der Lerneinheit haben sollen. Neben dem Bereich zum Einstieg in das Themengebiet besteht jede Nano-Lerneinheit noch aus einem Hauptbereich. Dieser umfasst die tatsächlichen Lerninhalte, welche in unterschiedlichen Formaten aufbereitet sind. Beispielweise bekommen die Studierenden einige Inhalte mittels Videos oder Podcasts präsentiert oder müssen sich anderes Wissen komplett selbst erarbeiten, in dem sie zum Beispiel einen wissenschaftlichen Artikel lesen und gemeinsam mit anderen Studierenden zusammenfassen. Jede Nano-Lerneinheit schließt mit einem kleinen Quiz zur Überprüfung des Gelernten ab und stellt darüber hinaus über Querverweise die Relevanz zu den anderen Inhalten her.

Prüfungsrelevant sind alle in Stud.IP Courseware bereitgestellten Nano-Lerneinheiten. Neben dem selbstständigen und intensiven Beschäftigen mit den einzelnen Nano-Lerneinheiten haben die Studierenden zusätzlich die Möglichkeit freiwillig an AC:DC teilzunehmen. Durch eine Teilnahme wird ein aktiveres und insbesondere durch den Aufbau des Konzepts ein kollaboratives und agiles Lernen digital gefördert. Wer sich dazu entscheidet daran mitzuwirken, hat die Chance auf einen Bonus in Form von Zusatzpunkten für die Klausur am Ende des Semesters. AC:DC ist von einem gemeinsamen Arbeiten geprägt, weshalb Gruppen aus jeweils vier bis sechs Studierenden zusammengestellt werden.

**Abb. 1:**
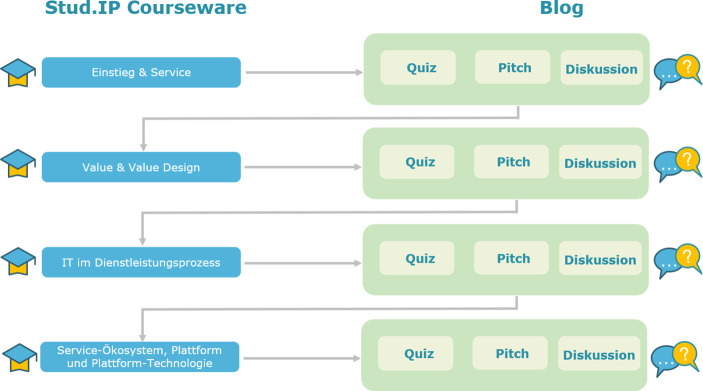
Ablauf von AC:DC

Jede Gruppe arbeitet praktisch und theoretisch die Inhalte der einzelnen Nano-Lerneinheiten anhand einer selbstgewählten Branche auf und beschäftigt sich dadurch intensiv mit den Besonderheiten dieser Branche. Lerninhalte werden somit beispielhaft durchdacht und hinterfragt. Im Anschluss an mehrere Nano-Lerneinheiten zu den Kernthemen Service, Value & Value Design, IT im Dienstleistungsprozess und Service-Ökosystem, Plattform & Plattform-Technologie (linker Teil der Abb. 1) folgt die Ausarbeitung eines **Pitches**, wobei jede Gruppe vor eine Herausforderung gestellt wird und diese möglichst innovativ lösen soll. Zum Beispiel soll zu dem Thema „Value & Value Design“ die selbstgewählte Dienstleistung aus dem ersten Pitch als eine perfekte Dienstleistung, ausgedrückt in servicedominierter-Logik, abgebildet werden. Dazu sollen Inhalte der zugehörigen Nanos bedacht und besonders auf die Werte eingegangen werden. Bei der Ausarbeitung des Pitches sind die Studierenden vollkommen frei und dürfen kreativ sein. Einige haben hierfür beispielsweise ein Video mit Legobausteinen gewählt, andere ein Comic gestaltet und wieder andere ganz klassisch eine kleine Präsentation vorbereitet. Bis zu einem bestimmten Termin sollen die Ergebnisse des Pitches auf dem Blog für die anderen Gruppen sichtbar veröffentlicht werden, so dass alle Studierenden die Lösungen der anderen kommentieren und bewerten können. Die Pitches werden anschließend einige Tage später noch in einer Videokonferenz vorgestellt und diskutiert. Der Blog bietet darüber hinaus zusätzlichen Raum zur **Diskussion**. Nicht nur die Pitches der anderen Gruppen können darauf besprochen werden. Zum Beispiel ist auch das Verlinken weiterführender Artikel oder das Stellen kritischer Fragen möglich. Ebenso ist es aber auch denkbar interessante, zusätzlich passende Beispiele vorzustellen. Zu jedem Kernthema ist auf dem Blog außerdem ein von den Lehrenden bereitgestelltes **Quiz** verfügbar, für dessen Bearbeitung die Studierenden bis zum Beginn der nächsten Lerneinheit Punkte erspielen können. Mit verschiedenen Aufgabentypen (z. B. Multiple-Choice oder Lückentext) wird hier erlerntes Wissen noch einmal überprüft. Abb. 1 visualisiert die Grundidee des Ablaufs der angepassten Vorlesung.

Durch die Alternierung von Selbstlernphasen, in denen jeder Studierende sein individuelles Lerntempo, durch die Nano-Lerneinheiten, verfolgen kann und den Interaktionsphasen, die durch Quizze, Pitches und Diskussionen bestimmt sind und bis auf den Präsentationstermin der Pitches der Selbstorganisation der Studierenden unterliegen, wird eine grobe Struktur vorgegeben, durch die der Wissenserwerb und der damit verbundene Aufwand möglichst gleichmäßig über das Semester verteilt ist.

**Abb. 2:**
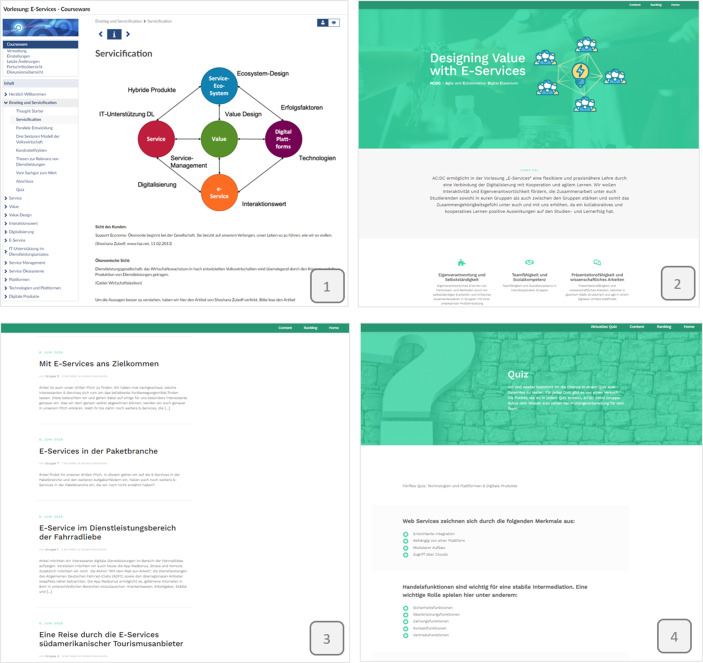
Screenshots aus Stud.IP Courseware und dem Blog

Abb. 2 zeigt beispielhafte Screenshots aus den verwendeten Systemen: Stud.IP Courseware und dem Blog. Im ersten Screenshot ist dabei ein Ausschnitt aus einer Nano-Lerneinheit zum ersten Kernthema „Einstieg und Service“ zu sehen. Des Weiteren zeigt der zweite Screenshot den Startbereich des Blogs, welcher mit Wordpress umgesetzt ist. Um Zugriff auf alle Inhalte zu erhalten, müssen sich die Studierenden anmelden. Die Authentifizierung ist mittels LDAP realisiert, damit die Studierenden mit ihren im Hochschulkontext gewohnten Anmeldedaten zugreifen können. Der dritte Screenshot visualisiert den Pitch- und Diskussionsbereich des Blogs. Hier stellen die Studierenden ihre Beiträge den anderen Teilnehmenden zur Diskussion zur Verfügung. Ein Ausschnitt aus dem Quiz zu dem vierten Kernthema „Service-Ökosystem, Plattform & Plattform-Technologie“ ist im vierten Screenshot zu sehen.

Für jede Beteiligung erhalten die Studierenden Punkte. Pitch-Beiträge bringen bis zu zehn Punkte, wobei der Durchschnitt aus den Bewertungen seitens der Studierenden und Lehrenden gebildet wird. Für allgemeine Blogbeiträge als Team oder Einzelperson gibt es automatisch drei Punkte und für Kommentare einen Punkt. Diese Punktevergabe soll sicherstellen, dass die Studierenden unmittelbar Feedback auf ihre Aktivität erfahren. Da die Qualität der Beiträge jedoch relevanter ist als die Quantität, korrigieren die Lehrenden gegebenenfalls die Punktzahl nach oben oder unten.

**Abb. 3:**
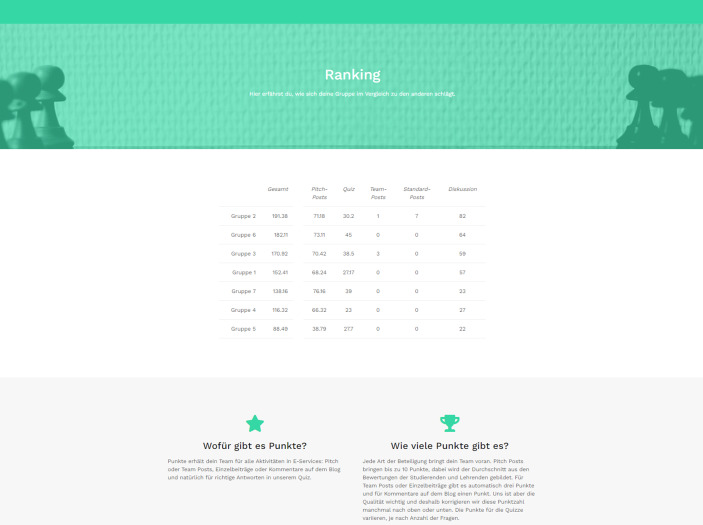
Screenshot des Gruppenrankings auf dem Blog

Je nach Anzahl an Fragen, variieren die erhaltenen Punkte für die Quiz-Teilnahme. Alle Punkte erarbeiten die Studierenden immer für die gesamte Gruppe, da das Gemeinsame den Fokus des Lehr- und Lernkonzepts bildet. Vor dem Hintergrund, dass Lernende unterschiedlich motiviert werden und einige bessere Lernergebnisse durch Kollaboration sowie Kooperation erzielen und andere wiederum mit Wettbewerbselementen (Kapp 2012), gibt es auf dem Blog neben den Punkten auch noch ein Gruppenranking. Dieses ist in Abb. 3 dargestellt. Jede einzelne Gruppe kann sich hierbei noch zusätzlich anzeigen lassen, für welche Beiträge und Kommentare wie viele Punkte vergeben worden sind. Dadurch ist Transparenz gewährleistet und es entsteht eine Nachvollziehbarkeit der Bewertungen.

### Der lehrveranstaltungsbegleitende Blog

Zur Unterstützung der Vorlesung „E-Services“ wird Stud.IP als digitale Umgebung schon seit vielen Jahren für die Bereitstellung der Lernmaterialien und zur Kommunikation zwischen Lernenden und Lehrenden verwendet. Das Aufarbeiten der Lerninhalte als einzelne Nano-Lerneinheiten in Stud.IP Courseware ist erst aufgrund von Covid-19 geschehen. Hierbei erfolgt neben der reinen digitalen Bereitstellung der Lerneinheiten auch eine Aufbereitung und Ergänzung durch multimediale Inhalte, wie zum Beispiel Erklärvideos und Podcasts, die entweder selbst produziert wurden oder aus anderen frei verfügbaren Quellen eingebunden wurden. Zur Aufrechterhaltung der vorhandenen und gewohnten hohen Interaktivität aus den Präsenzveranstaltungen ist die Entwicklung und Einführung des zusätzlichen lehrveranstaltungsbegleitenden Blogs ebenfalls bedingt durch Covid-19 geschehen.

Der Blog enthält ein eigens programmiertes AC:DC-Plugin, welches die verschiedenen Funktionen des Lehr- und Lernkonzepts unterstützt und für eine Nachnutzung geeignet ist. Im Administrationsbereich des Blogs haben die Lehrenden dadurch verschiedene Anpassungsoptionen.

Zunächst gibt es allgemeine Einstellungen. Dabei können Lehrende angeben, wann das Semester endet. Ab diesem Datum werden eingehende Beiträge und Kommentare auf dem Blog nicht mehr für das Ranking gewertet. Darüber hinaus können die automatisch vom System vergebenen Punkte als unmittelbares Feedback für jegliche Form der Aktivität festgelegt werden (z. B. 3 Punkte für Blogbeiträge und 1 Punkt pro Kommentar). Ein Eintragen der maximal möglichen Punktzahl für die Pitch-Beiträge ist ebenfalls möglich. In der derzeitigen Realisierung können Lehrende und Studierende einzelne Pitch-Beiträge jeweils mit maximal zehn Punkten bewerten.

Neben den allgemeinen Einstellungen ist auch die Verwaltung der Studierendengruppen und Lehrenden möglich. Neu eingeloggte Nutzer/-innen können somit zu Gruppen zusammengefasst werden und neue Lehrende können vollständige Zugriffsrechte erhalten.

Weiterhin sind Einstellungen bezüglich der eingebundenen Quizze möglich. Ein vorbereitetes Quiz kann in diesem Bereich ausgewählt und für einen bestimmten Zeitraum freigeschaltet werden. Das Quiz ist dann nur für den angegebenen Zeitraum verfügbar und wird auch nur währenddessen als Menüpunkt auf dem Blog angezeigt. Jedes Quiz ist dabei für die Studierenden nur einmal abrufbar. Das bedeutet, die einmal erreichten Punkte zählen für das Ranking und ein wiederholtes Probieren ist nicht möglich. Nach Ablauf des Zeitraums werden die Fragen und richtigen Antworten jedoch für alle dauerhaft sichtbar geschaltet, so dass ein Durchgehen noch einmal, vor allem in Hinblick auf die Prüfungsvorbereitung, unterstützt ist.

## Schlussbemerkungen

### Fazit

Die Vorlesung „E-Services“ wurde mit dem Lehr- und Lernkonzept AC:DC erstmals im Sommersemester 2020 (April bis Juli) durchgeführt. Insgesamt haben 40 Studierende aufgeteilt auf sieben Gruppen freiwillig daran teilgenommen. Durch eine erfolgreiche Teilnahme am AC:DC hatten die Studierenden die Möglichkeit sich einen Teil der erbrachten Leistungen (bis zu einem Teilnotensprung) als Vorleistung für die Klausur anrechnen zu lassen.

Die Lehrevaluationen haben gezeigt, dass die Studierenden die Veranstaltung gut bis sehr gut bewerten. Auf die Frage, was an der Lehrveranstaltung besonders gut gefallen hat und was noch verbessert werden könnte, gaben die Studierenden unterschiedliche Antworten. Beispielsweise heißt es in einem Kommentar „Das AC:DC war eine sehr gute Ergänzung zur Vorlesung. Die praktische Anwendung des Lernstoffes war sehr hilfreich“. Weiterhin ergänzt der Studierende, dass er es begrüßen würde, wenn weitere Lehrveranstaltungen das Konzept anwenden würden. In einem anderen Kommentar heißt es „Die Nano-Einheiten helfen dabei die Inhalte der Vorlesung zu verstehen und zu verinnerlichen. Sie sollten auf jeden Fall als Konzept beibehalten werden. Jedoch wäre es sehr hilfreich, wenn sie sich vom Aufbau mehr ähneln“. Bedingt durch Covid-19 und die sehr kurzfristige Übertragung und Realisierung der Vorlesungsinhalte als Nano-Lerneinheiten sowie der Tatsache, dass mehrere verschiedene Mitarbeitende des Lehrstuhls diese erarbeitet und eingepflegt haben, sind offensichtlich trotz einer festgelegten Struktur der einzelnen Nanos aus Thought-Starter, Haupt- und Schlussteil Unstimmigkeiten in der Strukturierung aufgetreten, die es gilt für nachfolgende Semester anzupassen. Ein weiterer Studierendenkommentar beinhaltet schlicht „Insgesamt eine gute Veranstaltung. :)“.

Neben dem positiven Feedback und den Hinweisen zur Verbesserung von den Studierenden zeigen auch die Noten aus der schriftlichen Klausur, wie das Lehr- und Lernkonzept gewirkt hat. An der Klausur haben 83 Studierende teilgenommen, wovon knapp die Hälfte aktiv an AC:DC partizipiert hat. Nachweislich haben die Studierenden, die sich bereits gemeinsam durchgängig im Semester mit den Lerninhalten beschäftigt haben, im Durchschnitt bessere Noten erreicht. Auffällig ist dabei vor allem, dass alle Studierenden, die eine Note im sehr guten Bereich (1,0–1,3) erreicht haben, an AC:DC teilgenommen haben.

Insgesamt lässt sich demnach festhalten, dass trotz des initialen, nicht unerheblichen, Aufwandes die vorhandenen Lerninhalte aufzubereiten sowie mit multimedialen Inhalten anzureichern und die digitale Plattform entsprechend des Lehr- und Lernkonzepts umzusetzen, das Lernerlebnis sowie das erworbene Wissen und damit zusammenhängend die Noten der Studierenden positiv beeinflusst wurden. Da der fortlaufende Aufwand für die Pflege und Aktualisierung der Inhalte und der Plattform jedoch nur einen Bruchteil des initialen Aufwands ausmachen, relativiert sich dieser in Anbetracht einer ggf. langjährigen Nutzung, so dass der Nutzen insbesondere bei längerer Verwendung, auch nach Covid-19, klar überwiegt.

### Ausblick

Vor allem aufgrund der positiven Resultate und Resonanz der Studierenden wird eine Verstetigung des Lehr- und Lernkonzepts AC:DC angestrebt. Während des Andauerns von Covid-19 wird AC:DC weiterhin eingesetzt und die angebotenen Lerninhalte werden erweitert sowie nachgebessert, um im Dialog mit den Studierenden qualitativ hochwertige Lerninhalte anzubieten und etwaige Unstimmigkeiten aufzulösen. Zudem wird geprüft, inwieweit sich das Lehr- und Lernkonzept AC:DC auf andere Lehrveranstaltungen sowohl innerhalb des Instituts als auch auf andere Institute übertragen lässt. Außerdem ermöglichen die Erfahrungen, welche bisher mit AC:DC gesammelt werden konnten und die noch in folgenden Semestern gesammelt werden eine umfassendere Evaluation des Lehr- und Lernkonzepts.

Auch nach Covid-19 ist ein weiterführender Einsatz von AC:DC geplant. Sobald Präsenzlehre wieder möglich ist, soll das Konzept AC:DC in den „normalen“ Lehrbetrieb, wie er vor Covid-19 stattgefunden hat, integriert werden, um die Vorteile, die beide Konzepte bieten, miteinander zu verbinden – freies, kollaboratives und selbstgesteuertes Lernen, gestützt durch eine digitale Plattform und die sozialen Nähe sowie Interaktion, wie sie nur eine Präsenzveranstaltung bieten kann.
